# BTApep-TAT peptide inhibits ADP-ribosylation of BORIS to induce DNA damage in cancer

**DOI:** 10.1186/s12943-022-01621-w

**Published:** 2022-08-02

**Authors:** Yanmei Zhang, Mengdie Fang, Shouye Li, Hao Xu, Juan Ren, Linglan Tu, Bowen Zuo, Wanxin Yao, Guang Liang

**Affiliations:** 1 School of Laboratory Medicine and Bioengineering, Hangzhou Medical College, Hangzhou, 310013 China; 2 College of Biotechnology and Bioengineering, Zhejiang University of Technology, Hangzhou, 310014 Zhejiang China; 3 Zhejiang Eyoung Pharmaceutical Research and Development Center, Hangzhou, 311258 Zhejiang China; 4 College of Pharmacy, Hangzhou Medical College, Hangzhou, 311300 Zhejiang China

**Keywords:** BORIS, Targeted peptide, ADP-ribosylation, DNA damage, Non-small cell lung cancer

## Abstract

**Background:**

Brother of regulator of imprinted sites (BORIS) is expressed in most cancers and often associated with short survival and poor prognosis in patients. BORIS inhibits apoptosis and promotes proliferation of cancer cells. However, its mechanism of action has not been elucidated, and there is no known inhibitor of BORIS.

**Methods:**

A phage display library was used to find the BORIS inhibitory peptides and BTApep-TAT was identified. The RNA sequencing profile of BTApep-TAT-treated H1299 cells was compared with that of BORIS-knockdown cells. Antitumor activity of BTApep-TAT was evaluated in a non-small cell lung cancer (NSCLC) xenograft mouse model. BTApep-TAT was also used to investigate the post-translational modification (PTM) of BORIS and the role of BORIS in DNA damage repair. Site-directed mutants of BORIS were constructed and used for investigating PTM and the function of BORIS.

**Results:**

BTApep-TAT induced DNA damage in cancer cells and suppressed NSCLC xenograft tumor progression. Investigation of the mechanism of action of BTApep-TAT demonstrated that BORIS underwent ADP ribosylation upon double- or single-strand DNA damage. Substitution of five conserved glutamic acid (E) residues with alanine residues (A) between amino acids (AAs) 198 and 228 of BORIS reduced its ADP ribosylation. Inhibition of ADP ribosylation of BORIS by a site-specific mutation or by BTApep-TAT treatment blocked its interaction with Ku70 and impaired the function of BORIS in DNA damage repair.

**Conclusions:**

The present study identified an inhibitor of BORIS, highlighted the importance of ADP ribosylation of BORIS, and revealed a novel function of BORIS in DNA damage repair. The present work provides a practical method for the future screening or optimization of drugs targeting BORIS.

**Supplementary Information:**

The online version contains supplementary material available at 10.1186/s12943-022-01621-w.

## Introduction

Brother of regulator of imprinted sites (BORIS) is frequently associated with malignant carcinomas and/or drug resistance [1, 2]. Almost all types of cancers express BORIS, including lung cancer, breast cancer, prostate cancer, and leukemia [3]. The Human Protein Atlas database collects pathology data from The Cancer Genome Atlas (TCGA), and the results of the analysis of these data showed a variable degree of correlation between elevated *BORIS* expression and shorter patient survival depending on the type of cancers (https://www.proteinatlas.org/ENSG00000124092-CTCFL/pathology). In contrast, BORIS expression is typically restricted to the testis and embryonic stem cells, but not in normal cells (https://www.proteinatlas.org/ENSG00000124092-CTCFL/tissue) [2, 4, 5]. Increased *BORIS* expression in carcinomas is usually due to demethylation of the *BORIS* promoter or copy number alterations of the *BORIS* gene [6–9]. Debruyne and colleagues reported elevated BORIS expression and its association with the development of resistance to ALK inhibition in neuroblastoma [1]. Because BORIS is expressed specifically in carcinomas but not in normal tissues, it can be applied for cancer diagnosis or therapy.

Immunizations with DNA encoding a BORIS antigen inhibited growth of mammary carcinomas and prolonged the survival of mice [2, 4, 10–12]. Cytotoxic T cell (CTL) immunotherapy targeting BORIS resulted in significant inhibition of cervical cancer progression and lung cancer cell proliferation [2, 12]. Although immunotherapies targeting BORIS showed curative effects in animal experiments, BORIS was not detected on the plasma membrane of cancer cells and immunotherapy targeting intracellular BORIS did not show the best result for clinical intervention. Because the structure of BORIS is not fully understood, it is not feasible to design inhibitors of BORIS based on its structure. To circumvent these problems, we employed a phage display library approach. The phage peptide library, which displays a variety of peptides, is suitable for the selection of potential inhibitory peptides against BORIS. In the present study, a specific region of BORIS was expressed and purified as an antigen to select an inhibitory peptide targeting BORIS.

The BORIS protein is composed of three parts: the N-terminal region, internal zinc finger-enriched region, and C-terminal region [5]. The internal zinc finger region of BORIS shares nearly identical zinc finger domains with its paralog CTCF (CCCTC binding factor) and is sufficient for nuclear localization [5, 13, 14]. BORIS and CTCF bind to the same DNA motif in vitro; however, these proteins share less than 40% of the common binding sites in the genome [15, 16]. Moreover, *BORIS* cannot substitute for *CTCF* deletion to sustain cellular activity [14]. We have previously reported that BORIS is located in both the cytoplasm and nucleus in cancer cells [5, 17, 18]; in contrast, CTCF is located only in the nucleus [14, 19]. BORIS promotes cancer cell growth, but CTCF suppresses cell growth. Therefore, the function of BORIS is substantially different from that of CTCF. The differences in the N-terminal and C-terminal regions may be responsible for their divergent functions [5, 14, 17, 20]. Twenty-three transcripts of *BORIS* are expressed from three alternative promoters using five distinct 5′ untranslated regions (UTRs) [21]. Different transcripts of *BORIS* are translated into six groups of proteins termed sf1 to sf6, all of them share a conserved N-terminal region [13]. The majority of commercial or reported antibodies to BORIS distinguish between normal and cancer tissues and are produced using antigens in the N-terminal region of BORIS [5, 17, 22]. These findings suggest that the N-terminal region of BORIS is specific to carcinomas cells and therefore useful for carcer diagnosis and suitable as an antigen for peptide selection.

Limited studies have reported the functional regions of BORIS. BAT3 interacts with the BORIS N-terminus at amino acids 1–50 to regulate *BRAC1* and *cMYC* expression [23]. Truncation of the internal zinc finger domains of BORIS yields a protein containing only N- and C-terminal regions, which is located in the cytoplasm and suppresses apoptosis of colorectal carcinoma [17]. The normal colon CCD-18Co cell line expresses truncated BORIS, which has intact 3–11 zinc finger domains and a C-terminal region and lacks the N-terminal region [17]. Knockdown of N-terminal-truncated BORIS in CCD-18Co cells did not affect cell growth [17]. BORIS sf6 is composed of an N-terminal region, five zinc finger domains, and a short unique C-terminal region. BORIS sf6 promotes the progression of cervical cancer and non-small cell lung cancer (NSCLC) [2, 12]. This observation indicates that BORIS proteins that contain the N-terminal region promote cancer development. However, a mutant that lacks the N-terminal region loses these functions, indicating that the N-terminus of BORIS may contain functional domains.

To select an inhibitory peptide targeting BORIS, the N-terminus of BORIS between amino acids 1 and 258 (BORIS-N_1-258_), which is different from its paralog CTCF, was used as an antigen for selection from the Ph.D.™-12 phage display peptide library. The peptide with the highest affinity was fused with the HIV-1 TAT peptide to confer ability to penetrate the cell membrane and was designated as BORIS-targeted peptide (BTApep-TAT). Validation of the effect of BTApep-TAT on NSCLC indicated that this inhibitor targeted BORIS. BORIS can repair both single- and double-strand DNA damage, but these functions were suppressed by BTApep-TAT. DNA breaks, radiation exposure, or hydrogen peroxide induced ADP-ribosylation of BORIS between amino acids 198 and 228. BTApep-TAT suppressed ADP ribosylation of BORIS, blocked the interaction of BORIS with Ku70, and attenuated DNA ligation induced by BORIS. The present study also developed a method for verification of the function of BORIS, which will benefit future drug screening or optimization.

## Materials and methods

### Construction of truncated BORIS and protein purification

The N terminal section of BORIS corresponding to AA 1–258 (BORIS-N_1-258_) and the N terminal-truncated BORIS (BORIS-del N_1-258_) were cloned by the Fast Mutagenesis System in the backbone of plasmid pFN6K. Both truncated BORIS constructs were fused with 6 consecutive histidine residues (His-tag) at the C-terminus for subsequent protein purification. Single step (KRX) competent cells (Promega Corporation) were used for protein expression. Proteins were purified by Ni–NTA column chromatography and confirmed by Western blotting using the anti-His antibody (Supplemental Fig. 1A). All plasmids used in this study are listed in Supplemental Table I.

**Fig. 1:**
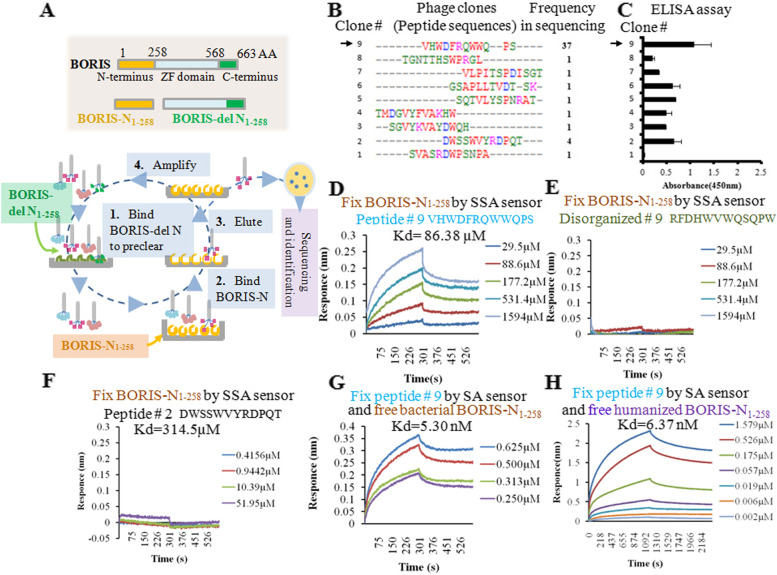
Selection and characterization of the BORIS-binding peptide. (**A**) Procedure for the selection of BORIS-binding peptides. (**B**) The sequences and frequencies of the peptides enriched after elution. (**C**) ELISA testing the affinity of phages for the BORIS-N_1-258_ protein. (**D**) The peptide from phage clone 9 was used to determine the affinity of the interaction with BORIS-N_1-258_ protein by BLI. The panel shows the test of the BORIS-N_1-258_ protein immobilized on an SSA sensor and free peptide in solution. (**E**) Scrambled peptide 9 showing no affinity to BORIS-N_1-258_ in the BLI assay. (**F**) Peptide 2 showed a weak binding affinity (Kd) of 314.5 µM to BORIS-N_1-258_ in the BLI assay. (**G**) Peptide 9 immobilized on an SA sensor and free BORIS-N_1-258_ protein in solution. (**H**) The BORIS-N_1-258_ protein purified from HEK293 cells was used to examine the interaction with synthesized peptide 9. The test was performed by fixing peptide 9 on an SA sensor and releasing the humanized BORIS-N_1-258_ protein to solution in a BLI assay

### Enrichment of phages from the random peptide display library

Ph.D.™-12 Phage Display Peptide Library Kit (New England Biolabs) was used for biopanning. This library contained 2 × 10^11^ unique 12-mer peptides linked to the N-terminus of a phage coat protein by a 4 amino acid spacer (GGGS). The library was diluted in TBS (50 mM pH 7.5 Tris–HCl, 150 mM NaCl). Microtubes coated with 100 µg/mL purified proteins in 0.1 M pH 8.6 NaHCO_3_ were used for the selection of phages. BORIS-del N_1-258_ was used in the first step of elutriation for 60 min at room temperature (RT) to remove non-specific phage clones. The pre-cleared phage library was used for further elutriations on BORIS-N_1-258-_coated microtubes for 60 min at room temperature and eluted with a general buffer (0.2 M pH 2.2 Glycine–HCl, 1 mg/mL BSA). The eluate enriched in BORIS-N_1-258_ binding phage clones was neutralized with 1 M pH 9.1 Tris–HCl. By two additional rounds of selection, phage clones enriched in BORIS-N_1-258_ binding clones were prepared.

### Test of selected phages in ELISA

The enriched phage clones were serially diluted and spread on Luria–Bertani agar plates. Sixty clones were randomly chosen for sequencing according to the manufacturer’s protocol. Nine peptide sequences were identified from the selected clones. We counted the frequency of each peptide displayed on the selected clones and examined the interactions between BORIS-N_1-258_ and the phage clones by ELISAs. ELISA was carried out as follows: ELISA plates (Thermo Scientific Nunc) were coated overnight at 4 ℃ with purified His-tagged BORIS-N_1-258_, BORIS-del N_1-258_ or BSA protein (10 mg/mL in carbonate buffer, pH 9.6), washed with PBST (0.1% Tween 20 in pH 7.4 PBS) three times and blocked with 5% BSA in pH 7.4 PBS for 1 h at RT. Purified phage clones were applied to the plates in serial dilutions (diluted with PBS containing 1% BSA) and incubated for 2 h at RT. After washing three times with PBST, bound phages were detected by a horseradish peroxidase-conjugated anti-M13 antibody (GE Healthcare, 27–9421-01) followed by incubation with the TMB substrate. Reactions were quenched using 250 mM HCl, and the absorbance at 450 nm was recorded by a plate reader (BioTek, Synergy 2).

### Examination of physical interaction between selected peptides and BORIS-N_1-258_

The candidate peptides that showed potential interactions with BORIS-N_1-258_ and the disorganized peptide were synthesized and conjugated with biotin or FITC by China Peptides Co., Ltd. (https://chinesepeptide.chemdrug.com/sell/). Twelve tandemly connected His was synthesized and conjugated with biotin or FITC as a negative control peptide by China Peptides Co., Ltd. The peptides were stored in powder at -80 °C. The peptides were dissolved in phosphate-buffered saline and filtered through a 0.22 µm filter to remove bacteria before use. The interaction between peptides and BORIS-N_1-258_ was measured by Bio-layer interferometry (BLI) in the ForteBio OctetRed system. The measurement was carried out in 5 steps: initial baseline duration, loading duration, baseline duration, association duration, and dissociation duration. In loading duration, 10 nM biotinylated peptides or 1 µM BORIS-N_1-258_ protein were immobilized on streptavidin-coated biosensors (ForteBio). The immobilization typically reached a response level of 4 nm. Association and dissociation curves were obtained through the addition of a dilution series of BORIS-N_1-258_ or peptides in PBS with 0.02% Tween 20 for the indicated period of time using Octet acquisition software. The binding data were fitted using Octet analysis software.

### Cell culture

H1299 cells (RRID: CVCL_0060) were cultured in RPMI 1640 medium supplemented with 10% FBS. HEK293 (RRID:CVCL_0045) and HeLa (RRID:CVCL_0030) cells were cultured in Dulbecco's modified Eagle’s medium (DMEM) supplemented with 10% FBS and placed in 37 ℃ and 5% CO_2_ incubators. Cells were seeded in 6-well plates at 1 × 10^5^ cells/well or in 96-well plates at 2000 cells/well for experiments. Cell viability was measured by MTT assay.

### Examination of interaction between selected peptides and BORIS-N_1-258_ in cells

The candidate peptides or negative control peptide fused with HIV-1 TAT sequence and conjugated with biotin were added to the cell lysate at a concentration of 25 µM and incubated for 24 h. Immunoprecipitation was performed to pull down peptides by streptavidin-conjugated magnetic beads (Cell Signaling Technology, 5947) and by BORIS primary antibody (Santa Cruz, CA, USA, sc-377085). Western blot assay was preformed after immunoprecipitation.

### Detection of cell viability and apoptosis induced by BTApep-TAT in vitro

Cells were plated and treated with BORIS-targeted peptide (BTApep-TAT) or negative control peptide (His-TAT) at concentrations of 10 µM, 25 µM, 50 µM and 100 µM for 3 days. Cell viability was measured by MTT assay. Cell apoptosis was measured by TUNEL assay (cat. # 40306ES20, Yeasen Biotech Co., Ltd) and Caspase-Glo 3/7 assay (G8090, Promega Corporation, an affiliate of Promega (Beijing) Biotech Co., Ltd.).

### RNA-sequencing analysis

Peptide-treated and siRNA-silenced H1299 cells were collected after three days of treatment. The sequences of the siRNAs used in this study are listed in Supplemental Table II. RNA was extracted using TRIzol (Invitrogen, Carlsbad, CA, USA) according to the manufacturer’s instructions. RNA from triplicate treated samples was purified and subjected to RNA-sequencing analysis using the DNBSEQ-G50 platform (BGI-Shenzhen, China). The differential gene expression analysis was performed by the Dr. Tom online system (BGI-Shenzhen, China, http://biosys.bgi.com). The heatmap was generated by Sangerbox 3.0 (http://vip.sangerbox.com/home.html). The BioProject accession is PRJNA832514. A few differentially expressed genes involved in the modulation of DNA damage regulation was validated by quantitative real-time PCR (qRT-PCR).

### Immunofluorescence assay

BTApep-TAT and BTApep were conjugated with biotin. The biotin-conjugated peptides were added to cell culture medium. Intracellular distributions of biotin-conjugated peptides were detected by Alexa Fluor® 488 Streptavidin (Yeasen Biotech Co., Ltd., Shanghai, China) and analyzed by an ImageXpress Micro Confocal system (Molecular Devices, LLC. San Jose, CA, USA).

### Quantitative real-time PCR

RNA was extracted from the cell pellet using TRIzol (Thermo Fisher Scientific) and ethanol precipitation. After quantification using a Nanodrop 2000 system, equal amounts of RNA from control and experimental samples were reversely transcribed into cDNA. The expression of candidate genes was quantified by qRT-PCR using *GAPDH* as a reference gene. The primers used in this study were reported in our previous study and are listed in Supplemental Table III [24].

### In vivo experiments in a mouse xenograft model

H1299 cells (1 × 10^6^ cells/injection) were subcutaneously injected into the limbs of NOD/SCID/γc null (NSG) mice. In this study, all animals were male. One week after cell inoculation, BTApep-TAT/His-TAT (dissolved in PBS) was injected intraperitoneally at 16 mg/kg body weight every other day for 3 weeks. Tumor volumes were recorded every other day. The weight and volume of the tumors were recorded at the end after surgical resection. Serum was collected from each mouse and used for liver (ALT, AST, ALP, DBIL, TBIL) and kidney function (CRE, UA) analyses by Servicebio Wuhan, China (https://www.servicebio.cn/). Tumors were sliced and examined by TUNEL assay by Servicebio. All experimental protocols were approved by the licensing committee of Hangzhou Medical College, P. R. China.

### Preparation of nuclear extract

Twenty million (2 × 10^7^) HeLa or H1299 cells were trypsinized, collected by centrifugation (2000 g, 5 min) and rinsed twice in PBS. Cell pellets were resuspended in a fivefold packed cell volume of hypotonic buffer (10 mM HEPES–KOH, pH 7.5 at 4 °C, 5 mM KCl, 1.5 mM MgCl_2_, 0.2 mM PMSF, 0.5 mM DTT), kept on ice for 10 min, and centrifuged (10 min, 1200 g). The cell pellets were resuspended in an equal volume of hypotonic buffer and disrupted in a Dounce homogenizer (20 strokes, pestle B). Subsequently, 3 M KCl was slowly added to a final concentration of 50 mM KCl, and the mixture was kept on ice for 10 min and centrifuged (3000 g, 20 min) to precipitate the nuclei. The nuclear pellets were resuspended in 2 packed nuclear volumes of low salt buffer (20 mM HEPES, pH 7.9 at 4 °C, 1.5 mM MgCl_2_, 20 mM KCl, 0.2 mM EDTA, 0.2 mM PMSF, and 0.5 mM DTT) and added to 1 volume of high salt buffer. Nuclear proteins were extracted at 4 °C for 30 min under gentle agitation and centrifuged at 10,000 × g for 30 min at 4 °C.

### In vitro assay for detecting DNA single-strand break repair (SSBR) by qRT-PCR

SSBR activity was analyzed by qRT-PCR, as described previously [25]. Crude nuclear extracts were isolated, and the protein content was quantified by the Bradford assay. Twenty-four micrograms (24 µg) of nuclear protein were incubated for 30 min at 32 ℃ in a 20 µl reaction mixture that contained 45 mM HEPES–KOH, 70 mM KCl, 7.4 mM MgCl_2_, 0.9 mM DTT, 0.4 mM EDTA, 2 mM ATP, 20 µl each of dATP, dTTP, cCTP, and dGTP, 40 mM phosphocreatine, 2.5 µg of creatine phosphokinase, 20 µg/ml BSA, 3.4% glycerol, 2 mM NAD + , 4 µg of poly(dI/dC), and templates listed in Supplemental Fig. 2 (2 pmol DNA template A, which had a break, or template B, which contained a single nucleotide deletion, or template C, which was endogenous control DNA). The reaction was terminated by heating at 72 °C for 10 min. Two microliters of 10,000 × dilution of the reaction mixture was used as a template for qRT-PCR. The probes and primers were designed as described [25]. The final concentrations of the forward and reverse primers were 200 nM. The final concentration of either probe was 300 nM. qRT-PCR monoplex reactions were performed by annealing at 60 °C for 40 cycles. The quantity of repaired templates was calculated by comparing the Ct values of repaired templates and control template.

**Fig. 2:**
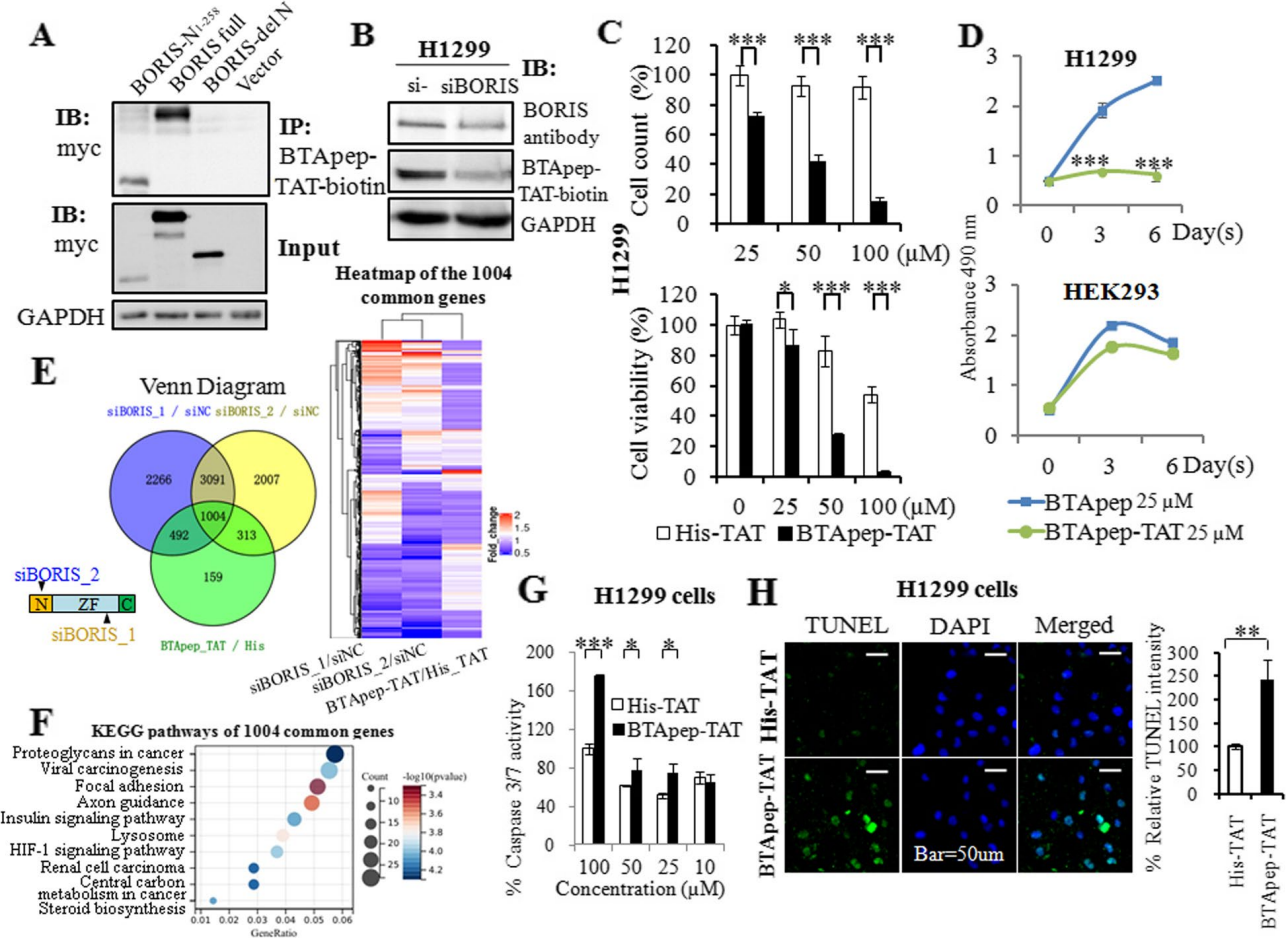
BTApep-TAT induced DNA damage and cancer cell apoptosis. (**A**) Co-immunoprecipitation was performed to evaluate the interaction of BTApep-TAT-biotin with BORIS-N_1-258_, full length BORIS, and BORIS-del N_1-258_ in the cell lysates from transfected H1299 cells. (**B**) The level of BORIS in H1299 cells after siRNA-mediated knockdown was evaluated by BORIS antibody or the BTApep-TAT-biotin peptide. (**C**) Cells were incubated with graded concentrations of the peptides (25–100 µM) for three days. MTT assays and cell counting were performed to evaluate the effect of BTApep-TAT and the negative control peptide His-TAT on H1299 cells. (**D**) H1299 and HEK293 cells were treated with 25 µM BTApep-TAT or BTApep to examine the effect of BTApep-TAT on cancer cells and normal cells (**E**) Transcriptomes of H1299 cells with siBORIS knockdown or BTApep-TAT treatment were compared. The left panel shows an overlap between siBORIS knockdowns and BTApep-TAT treatment in a Venn diagram. Two siRNAs targeting *BORIS* were used to compare the common genes in the heatmap. (**F**) A bubble map showing the pathways associated with the genes common to BORIS knockdown and BTApep-TAT treatment, which are shown in Panel E. (**G**) Caspase 3/7 assay detected the peptide-induced apoptosis at peptide concentrations from 10 to 100 µM. (H) A TUNEL assay detected the DNA damage induced by 25 µM peptide

### In vitro end-joining assay

A plasmid-based assay for in vitro end joining was performed as described [26]. The end-joining reaction was performed in a final volume of 30 µl by incubating 100 ng of *Xho*I-digested pCMV6 plasmid (RRID:Addgene_58320) with 10 µg of nuclear extract from HeLa cells for 1 h at 25 °C in NHEJ buffer (20 mM HEPES–KOH, pH 7.5 at RT, 80 mM KCl, 10 mM MgCl_2_, 1 mM ATP, 1 mM DTT, 50 mM dNTP, 80 mM NaCl and protease inhibitors). The reaction was stopped with 2 µl of 0.5% SDS, 2 µl of 0.5 M EDTA, followed by 1 µl proteinase K (10 mg/mL) treatment at 37 °C for 0.5 h. Ten microliters of the samples were separated by running on a 0.7% agarose gel at 2 V/cm in 0.5 × TBE buffer. The gel was stained with SYBR Gold I (diluted 1:20,000 in 0.5X TBE) and visualized under UV light.

### Fluorescence-based DNA repair assay in cells

Alternative NHEJ (RRID:Addgene_44025), total NHEJ (RRID:Addgene_44026), and DSB Repair (Homology directed repair) DNA repair report (RRID:Addgene_26475) systems were used to investigate the function of BORIS in cells [27]. BORIS-RFP plasmid with pCMV6-Entry backbone and fluorescent-based DNA repair reporter plasmids were co-transfected into HeLa cells for 24 h. Flow cytometry was used to count and analyze the cells with red and/or green fluorescence. The cells expressing only GFP or RFP were used as controls. The cells expressing both red and green fluorescence indicated that BORIS-RFP was expressed and that DNA damage was successfully repaired. The cells without red fluorescence were used to analyze spontaneous DNA repair. The percentage of cells that underwent DNA repair was determined by calculating the percentage of cells with GFP fluorescence in BORIS-RFP-transfected cells or cells without transfection (or without RFP fluorescence). The proportion of cells with GFP fluorescence was compared between cells with and without RFP fluorescence.

### Detection of ADP ribosylation of BORIS

HeLa or H1299 cells were lysed in immunoprecipitation (IP) buffer (50 mM Tris–HCl pH 7.4, 150 mM NaCl, 1% NP-40, 1 mM EDTA, 5% glycerol) containing protease inhibitor cocktails. The cell lysates were incubated with mouse anti-Myc (CST, 2276) or rabbit anti-poly/mono-ADP Ribose (E6F6A) monoclonal antibody (CST, 83732) on a rotary shaker at 4 °C overnight. Mouse or rabbit IgG (Santa Cruz, sc-2025 or sc2027) was used as a negative control for detecting the pull-down specificity. Protein G beads (Santa Cruz, sc-2001) or protein A beads (Santa Cruz, sc-2002) were added and incubated at room temperature for 2 h. The agarose beads were collected by centrifugation, washed five times with IP buffer according to the manufacturer’s instructions, and eluted in SDS sample buffer for the subsequent Western blotting assay. Olaparib (Selleck, S1060) was purchased from Selleck Chemicals. The instrument used for X-ray irradiation was a RAD SOURCE RS 2000pro-225 X-RAY IRRADIATOR.

### In vitro ADP-ribosylation assay

Dynabeads™ M280 Streptavidin Beads (Invitrogen, 60,210) immobilized with 5 pmol biotin-PAR polymers (Trevigen, 4336–100-01) or empty Streptavidin beads were incubated together with 12.5 to 100 pmol of purified BORIS protein in NETN buffer (50 mM Tris–HCl pH 8.0, 100 mM NaCl, 2 mM EDTA, 0.5% NP-40). After incubation for 1 h at room temperature, beads were washed with NETN buffer 5 times, and bound proteins were released by adding 30 µl SDS sample buffer followed by heating at 90 °C for 10 min for the subsequent Western blotting assay.

### PARP1-catelyzed in vitro poly ADP ribosylation (PARylation) assay

Peptides (1–4 μg) incubated with different samples were added to 50 μL PARP1 reaction buffer (50 mM Tris–HCl at pH 7.4, 2 mM MgCl_2_, 200 μM NAD^+^), which contained 0.2 μg of recombinant PARP1 (Trevigen, 4668–100-01) and 2.5 μg of ssDNA (Sigma, D8899), and reaction was carried out at 37 °C for 30 min. Low molecular weight peptide and PARP1 protein in the reactions were separated by a 30 kDa cutoff centrifugal filter (Millipore, UFC803096). Then, SDS loading buffer was added to separate peptides or proteins and analyzed by dot blotting using an anti- ADP ribosylation (ADPr) antibody.

### Antibodies and co-immunoprecipitations

The BORIS antibody was purchased from Santa Cruz Biotechnology (Santa Cruz, sc-377085). Streptavidin-conjugated magnetic beads (CST, 5947), Myc-Tag (CST, 2276) mouse monoclonal antibody, and poly/mono-ADP ribose (E6F6A) rabbit monoclonal antibody (CST, 83,732) were purchased from Cell Signaling Technology, (Danvers, MA). Anti-mono-ADP ribose recombinant antibody (Sigma, MABE1076) was purchased from Sigma. Anti-poly-ADP ribose monoclonal antibody (Trevigen, 4335-MC-100) was purchased from Trevigen. All antibodies used in this study are listed in Supplemental Table IV. The materials of other chemicals and reagents are listed in Supplemental Table V.

### Statistical analysis

All data were obtained in a minimum of triplicates and are expressed as the mean ± standard deviation (SD). Statistical differences between the controls and treatments were evaluated by two-tailed Student’s t test. *P* < 0.05 was considered statistically significant.

## Results

### Selection and characterization of the BORIS-binding peptide

BORIS N-terminal region AA 1–258 (BORIS-N_1-258_) and BORIS N-terminal deletion (BORIS-del N_1-258_) were used to enrich the BORIS-N_1-258_-binding phage clones by alternately eluting from the Ph.D.™-12 phage display peptide library (Figs. 1A and Supplemental Figs. 1A). BORIS-del N_1-258_ was used to remove non-specific phage clones, and BORIS-N_1-258_ was used for subsequent phage enrichment. After four rounds of enrichment, the titer of the eluted phages reached a plateau (Supplemental Fig. 1B). Sixty phage clones were randomly selected for sequencing the displayed peptides. Nine peptides were identified in the sequenced clones, and peptide 9 was the most frequent one (Fig. 1B). Then, the enriched phage clones for these nine peptides were tested by ELISA using BORIS-N_1-258_-coated plates (Fig. 1C). Phage clone 9 had the highest binding affinity for the BORIS-N_1-258_ protein_._

The displayed peptides that were found in multiple clones (#9 and #2) or had high binding affinity (#9) were synthesized to assay their physical interaction with the BORIS-N_1-258_ protein by Bio-layer interferometry (BLI) using a Fortebio Octet RED system. The BORIS-N_1-258_ protein was conjugated with biotin and loaded onto an SSA sensor. The synthesized peptides were dissolved in PBS with 0.02% Tween 20. Peptide 9 (the peptide sequence was VHWDFRQWWQPS) bound to BORIS-N_1-258_ with a strong binding affinity (Kd) of 86.38 µM (Fig. 1D). Scrambled peptide 9 (the peptide sequence was RFDHWVWQSQPW) did not have any measurable affinity to BORIS-N_1-258_ (Fig. 1E). Peptide 2 (DWSSWVYRDPQT) bound to BORIS-N_1-258_ with a weak binding affinity (Kd) of 314.5 µM (Fig. 1F). To confirm the interaction by a reciprocal experiment, peptide 9 was labeled with biotin, loaded onto an SA sensor and tested using a series of concentrations of BORIS-N_1-258_ to determine the Kd value, which was shown to be 5.30 nM (Fig. 1G).

The BORIS-N_1-258_ antigen used to select peptide 9 was purified from the bacteria. To confirm the interaction between peptide 9 and BORIS-N_1-258_ in human cells, BORIS-N_1-258_ was expressed and purified from HEK293 cells, which do not express *BORIS*. Peptide 9 bound to human cell-derived BORIS-N_1-258_ with a Kd value of 6.37 nM, which was comparable to the affinity of peptide 9 to the BORIS antigen expressed in bacteria (Kd = 5.30 nM) (Fig. 1G and 1H).

### The selected peptide specifically bound to BORIS in cells

A fusion peptide of peptide 9 and the HIV-1 TAT peptide (the peptide sequence was GGRKKRRQRRRG) was synthesized and labeled with biotin. Fusion with the HIV-TAT peptide confers ability to penetrate the cell membrane. Biotinylated peptide 9 fused with HIV-1 TAT was designated as BTApep-TAT-biotin in the present study. A peptide that lacked the TAT penetrating peptide and labeled with biotin (BTApep-biotin) was synthesized to evaluate cell membrane penetration efficiency (Supplemental Fig. 1C). A biotinylated peptide containing twelve consecutive histidines fused with HIV-1 TAT peptide was used as a negative control peptide (His-TAT-biotin). Myc-tagged BORIS and two truncated constructs were overexpressed in H1299 cells. The cell lysate was collected and incubated with 25 µM BTApep-TAT-biotin to evaluated the interaction between BTApep-TAT-biotin and BORIS by co-immunoprecipitation. BTApep-TAT-biotin was shown to bind to the full-length BORIS and BORIS-N_1-258_ but not BORIS-del N_1-258_ (Fig. 2A). Upon *BORIS* knockdown in H1299 cells by siRNA (siBORIS_1), the binding of both a commercial BORIS antibody (Santa Cruz; sc-377085) and the BTApep-TAT-biotin peptide decreased, suggesting that BTApep-TAT was able to specifically bind to BORIS in the cells (Fig. 2B). Next, the cells were cultured with BTApep-TAT-biotin or BTApep-biotin for 20 h, and the localization of the peptides were analyzed by immunofluorescence. It was found that BTApep-TAT-biotin, but not BTApep-biotin, penetrated into the cells (Supplemental Fig. 1C).

### BTApep-TAT induced DNA damage and apoptosis in cancer cells

BTApep-TAT was able to bind to BORIS, which plays important roles in cancer development [1, 4, 17, 24]; hence, we tested the effect of the peptide on the proliferation and apoptosis of carcinoma cells. H1299 lung cancer cells were treated with graded dilutions of BTApep-TAT, and MTT assays and cell counting were performed. It was found that BTApep-TAT, but not the His-TAT control, suppressed the proliferation of H1299 cells at concentrations of 25–100 µM after 3 days of treatment (Fig. 2C). Additionally, BTApep-TAT suppressed the proliferation of H1299 cells but not normal HEK293 cells, which do not express BORIS (Fig. 2D). BTApep did not inhibit cell proliferation because it did not penetrate through the cell membrane (Fig. 2D and Supplemental Fig. 1C).

To investigate the targeting specificity of BTApep-TAT toward BORIS, the transcriptomes of H1299 cells treated with BTApep-TAT and those subjected to *BORIS* knockdown by two siRNAs were compared by high-throughput RNA sequencing. Heatmap comparison of the gene expression profiles among BTApep-TAT treatment and BORIS knockdown samples is presented (Fig. 2E). The 1004 common genes are listed in Supplemental Table VI. KEGG pathway analysis by Sangerbox 3.0 (http://vip.sangerbox.com) showed the top pathways associated with the 1004 common genes (Fig. 2F). In H1299 cells, BTApep-TAT induced apoptosis as shown by the results of the caspase-3/7 assay and caused DNA damage as shown by the results of the TUNEL assay (Fig. 2G and 2H). BORIS prevents cancer cells from undergoing apoptosis and protects genomic stability, as shown in our previous studies [17, 24, 28]; hence, BTApep-TAT treatment may attenuate the protective effect of BORIS on cancer cell genome stability.

### BTApep-TAT inhibited DNA damage repair governed by BORIS in cancer cells

**Fig. 3:**
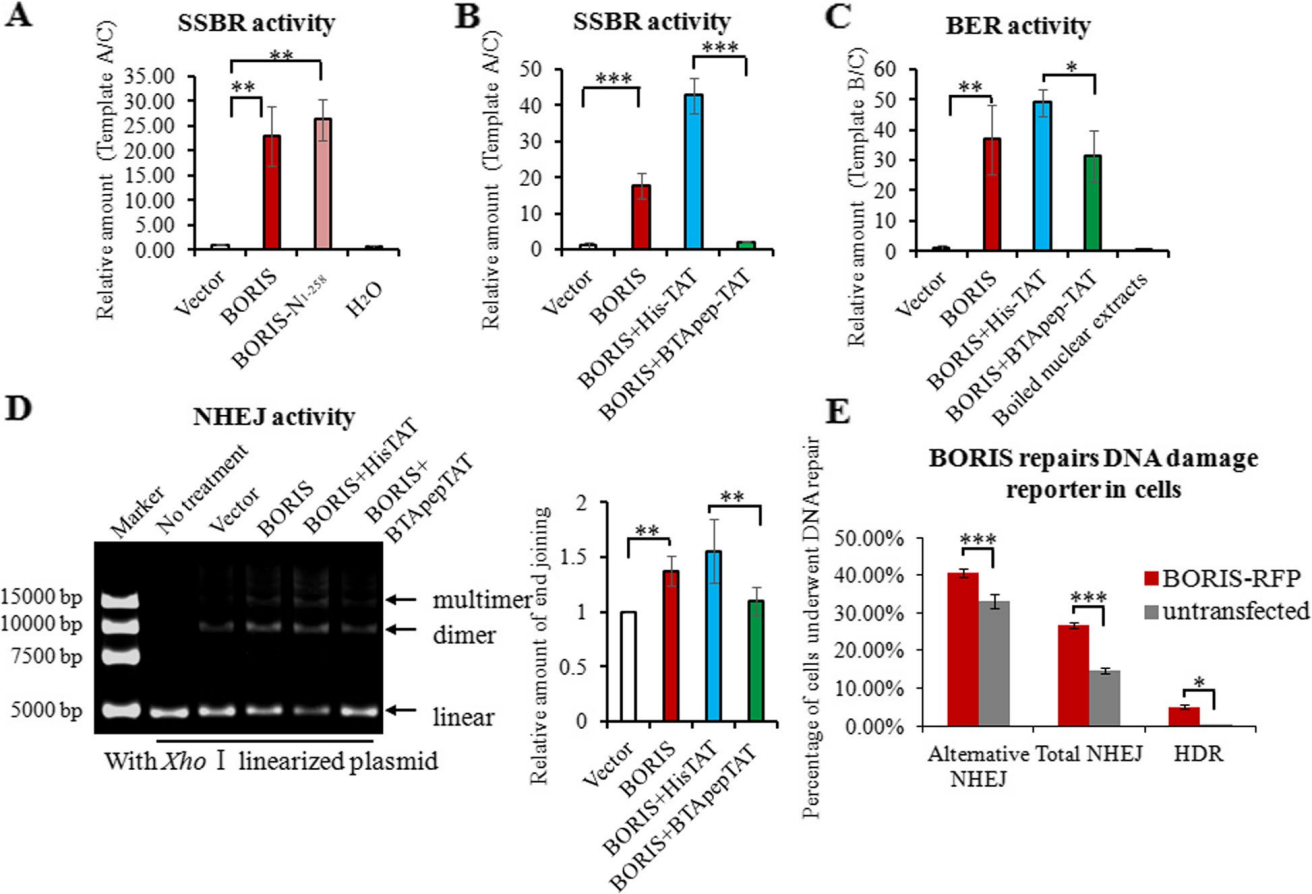
BTApep-TAT inhibited DNA damage repair governed by BORIS in cancer cells. (**A**) Artificial single-strand break DNA was incubated with crude nuclear extracts from HeLa cells transfected with full-length BORIS or the AA 1–258 N-terminus of BORIS (BORIS-N_1-258_). Crude nuclear extracts of empty vector-transfected cells or equal volumes of water were used as negative controls. The DNA repair activities were examined by qRT–PCR and are shown as the relative transcript amount of ligated target A templates. (**B**) SSBR activities were examined in BORIS-expressing crude nuclear extracts that were treated with 25 µM peptides. (**C**) BER activities were examined in BORIS-expressing crude nuclear extracts that were treated with 25 µM peptides. The boiled nuclear extracts from BORIS-expressing HeLa cells were used as another negative control. The DNA repair activities were examined by qRT-PCR and are shown as the relative transcript amount of ligated target B templates. (**D**) *The Xho* I-linearized plasmid was used as double-strand DNA break to evaluate NHEJ activity. The multimeric and dimeric forms of the plasmids were visualized on agarose gels, which are presented in the left panel. The right panel shows the statistical summary of the treatment results. (**E**) DNA damage repair reporters (GFP fluorescent-based DNA repair reporter system) were used to evaluate the effect of BORIS in cells. The percentage of cells that underwent DNA damage repair was compared between BORIS-RFP-transfected cells and cells without transfection. DNA damage repair, which includes alternative NHEJ, total NHEJ, and homology-directed repair (HDR), was analyzed by flow cytometry

Environmental and internal stresses, such as mutagenic chemicals, ionizing radiation (IR), reactive oxygen species (ROS), and mis-replication stress, induce DNA lesions, including DNA single-strand breaks (SSBs) and double-strand breaks (DSBs). Impairment of DNA repair accumulates DNA lesions and results in genomic instability. Abnormal DNA repair promotes cancer progression [29]. Previously, we showed that BORIS promoted DNA repair in NSCLC cells [24]. In the present study, we found that BTApep-TAT induced DNA damage and attenuated the protective effect of BORIS on the cancer cell genome. To evaluate single-strand break repair (SSBR) and base excision repair (BER) activities in vitro, the quantity of the ligation products of the target templates was measured. The target templates were the DNA break fragments or fragments with single nucleotide deletion, and the control template was intact DNA without breaks (Supplemental Fig. 2A). The primers and probes used to measure the ligation efficiency of the target templates are listed in Supplemental Fig. 2B. A mixture of the target and endogenous control templates was used to evaluate the effects of the treatments on DNA repair in the present study. Crude nuclear extracts of HeLa cells transfected with full-length BORIS or BORIS-N_1-258_ were incubated with a mixture of the templates containing DNA lesions and control templates in a cell-free assay. BORIS-N_1-258_ enhanced SSBR to the same extent as BORIS (Fig. 3A), indicating that the AA 1–258 N-terminus of BORIS was responsible for the SSBR activity of BORIS. Next, BTApep-TAT or His-TAT (negative control peptide) was added to the crude nuclear extracts of BORIS-expressing HeLa cells. BTApep-TAT significantly inhibited SSBR and BER but not His-TAT (Fig. 3B and 3C). These results were consistent with the finding that BTApep-TAT, but not His-TAT, induced DNA damage and apoptosis in cancer cells (Fig. 2G and 2H).

In addition to DNA single-strand damage, the repair of DNA double-strand breaks (DSBs) is common in mammalian cells. As nonhomologous end joining (NHEJ) is an efficient mechanism of DSB repair and prevalent in animal cells [30, 31]. We evaluated the functions of BORIS and BTApep-TAT in NHEJ using the *Xho* I-linearized plasmid-based in vitro DNA end-joining assay. Nuclear extracts from BORIS-expressing HeLa cells promoted the generation of the multimeric and dimeric forms of the plasmids visualized on agarose gels. The data indicated that BORIS promoted efficient end joining. BTApep-TAT, but not His-TAT, treatment inhibited BORIS-induced end joining by approximately 30% (Fig. 3D). The above experiments were performed in cell-free assays by mixing crude nuclear extracts and artificial DNA segments. To validate the effect of BORIS in cells, GFP fluorescence-based DNA repair reporter experiments were performed [27]. The percentage of cells that underwent DNA damage repair was compared between BORIS-RFP-transfected cells and cells without transfection. Although a proportion of cells have spontaneous DNA damage repair, the introduction of BORIS-RFP promoted all kinds of DNA damage repair, including alternative NHEJ, total NHEJ, and HDR (homology-directed repair) (Fig. 3E). Flow cytometry was used to count and analyze the cells. Overall, results from these experiments indicated that BORIS promoted the repair of both SSBs and DSBs in cancer cells. The inhibitory peptide BTApep-TAT suppressed DNA damage repairs that were governed by BORIS.

### BTApep-TAT inhibited the ADP ribosylation of BORIS in response to DNA damage

**Fig. 4:**
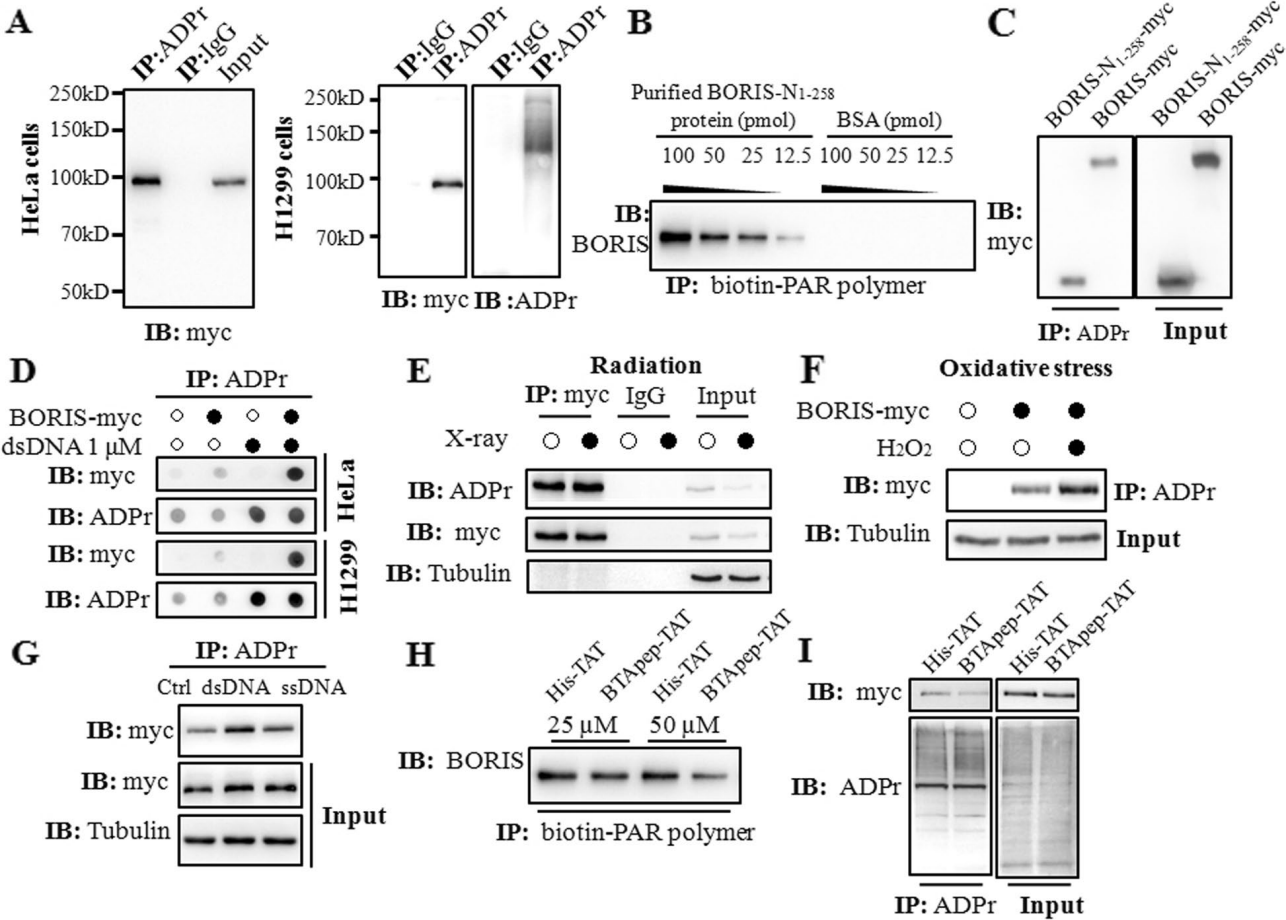
BTApep-TAT inhibited the ADP ribosylation of BORIS in response to DNA damage. (**A**) ADP-ribosylation of BORIS was determined in BORIS-myc-transfected HeLa and H1299 cells using the ADPr antibody, which detected both poly-ADPr and mono-ADP-ribosylation. (**B**) The purified BORIS-N_1-258_ protein or BSA was diluted and incubated with 5 pmol of biotin-PAR polymers immobilized on streptavidin beads. Specific interaction was observed with the BORIS-N_1-258_ protein, but not with BSA. (**C**) ADP-ribosylation of BORIS-N_1-258_-myc was determined in transfected H1299 cells. The levels of ADP ribosylation were nearly identical for BORIS-N_1-258_ and full-length BORIS. (**D**) The plasmids of BORIS-myc or empty vector were transfected into H1299 and HeLa cells. The crude nuclear extracts were supplemented with 1 μM dsDNA. BORIS was ADP-ribosylated in both H1299 and HeLa cells, and ADP-ribosylation was enhanced upon dsDNA induction. (**E**) DNA damage was induced in H1299 cells by 30 Gy X-ray irradiation. (**F**) DNA damage was induced in H1299 cells by treatment with H_2_O_2_ at a concentration of 500 µM for 10 min. (**G**) The levels of ADP ribosylation of BORIS-myc in H1299 cells were compared between dsDNA and ssDNA treatments. (**H**) ADP ribosylation of BORIS-N_1-258_ was examined by an in vitro ADP-ribosylation assay. BTApep-TAT treatment significantly suppressed ADP ribosylation of BORIS-N_1-258._ (**I**) ADP ribosylation of BORIS-myc in H1299 cells after treatment with 25 µM BTApep-TAT or His-TAT was examined by immunoprecipitation of ADP-ribosylated protein and immunoblotting against the myc tag

HeLa and H1299 cells were transfected with C-terminal Myc-tagged BORIS. Immunoprecipitation with an anti-ADP-ribose antibody (recognizes both poly- and mono-ADP-ribosylation) indicated that BORIS-myc was modified by ADP-ribose (Fig. 4A). To confirm ADP ribosylation of BORIS, an in vitro ADP ribosylation assay was performed using the purified BORIS-N_1-258_ protein and biotin-labeled PAR polymers. Biotin-labeled PAR bound to BORIS-N_1-258_ in a concentration-dependent manner in vitro (Fig. 4B). Next, H1299 cells transfected with BORIS-N_1-258_-myc were immunoprecipitated with an anti-ADP-ribose antibody, and the modification of BORIS-N_1-258_-myc was confirmed. The ADP-ribosylation level of BORIS-N_1-258_-myc was comparable with that of full-length BORIS (BORIS-myc) (Fig. 4C). These results demonstrated ADP ribosylation of BORIS in amino acids 1–258.

Next, the ADP-ribosylation level of BORIS was investigated under DNA damage conditions. Synthetic double-strand DNA (dsDNA) was used to mimic DNA breaks in the cells undergoing DNA damage. Synthetic dsDNA was added to the lysate of BORIS-expressing cells, and the ADP-ribosylation level was examined. dsDNA promoted ADP ribosylation of BORIS in HeLa and H1299 cells (Fig. 4D). Furthermore, IR (irradiation) slightly induced ADP ribosylation of BORIS (Fig. 4E), and H_2_O_2_ induced considerable ADP ribosylation of BORIS (Fig. 4F). X-ray irradiation produces 25-fold more SSBs than DSBs [32]. These results suggested that there was a difference in the magnitude of ADP-ribosylation of BORIS induced by dsDNA or ssDNA. Although both ssDNA and dsDNA promoted ADP ribosylation of BORIS, dsDNA showed stronger induction than ssDNA (Fig. 4G).

Additionally, we assessed the type of ADP ribosylation of BORIS. Two antibodies that distinguished mono-ADPr and poly-ADPr and a broad specificity antibody detecting both mono-ADPr and poly-ADPr were used. BORIS was modified by both poly-ADPr and mono-ADPr upon DNA damage (Supplemental Fig. 3A). Incubation with the PARP inhibitor olaparib did not attenuate ADP ribosylation of BORIS (Supplemental Fig. 3B). To identify the polymerase which catalyzes the ADP-ribosylation of BORIS, PARPs were knocked down by siRNAs individually. Knockdown of *PARP1*, *PARP4*, *PARP5a*, *PARP8*, *PARP13* and *PARP16* inhibited ADP ribosylation of BORIS (Supplemental Fig. 3C). Although PARP1 could be inhibited by olaparib [33], catalysis by PARP4, PARP5a, PARP8, PARP13 and PARP16 could not be abolished by olaparib treatment. The ADP-ribosylation rate on BORIS was investigated by the treatment of the mixture of single- and double-strand DNA for 30, 60, and 90 min. The ADP-ribosylation peaked at 30 min of treatment and declined after 60 min (Supplemental Fig. 3D). The rapid ADP ribosylation of BORIS reflects the significance of BORIS in DNA damage repair.

To determine whether BTApep-TAT influences ADP ribosylation of BORIS, the purified BORIS-N_1-258_ protein was preincubated with 25 µM and 50 µM BTApep-TAT or His-TAT before the in vitro ADP-ribosylation assay was performed. The results indicated that BTApep-TAT efficiently inhibited ADP ribosylation of BORIS-N_1-258_ (Fig. 4H). Modification of BORIS-myc transfected into H1299 cells was also suppressed by 25 µM BTApep-TAT treatment but not by His-TAT treatment (Fig. 4I). These results showed that ADP ribosylation of BORIS was markedly inhibited both in vitro and in vivo by BTApep-TAT.

### BTApep-TAT inhibited the ADP ribosylation in residues 198–228 of BORIS, and blocked the interaction of BORIS and Ku70

**Fig. 5:**
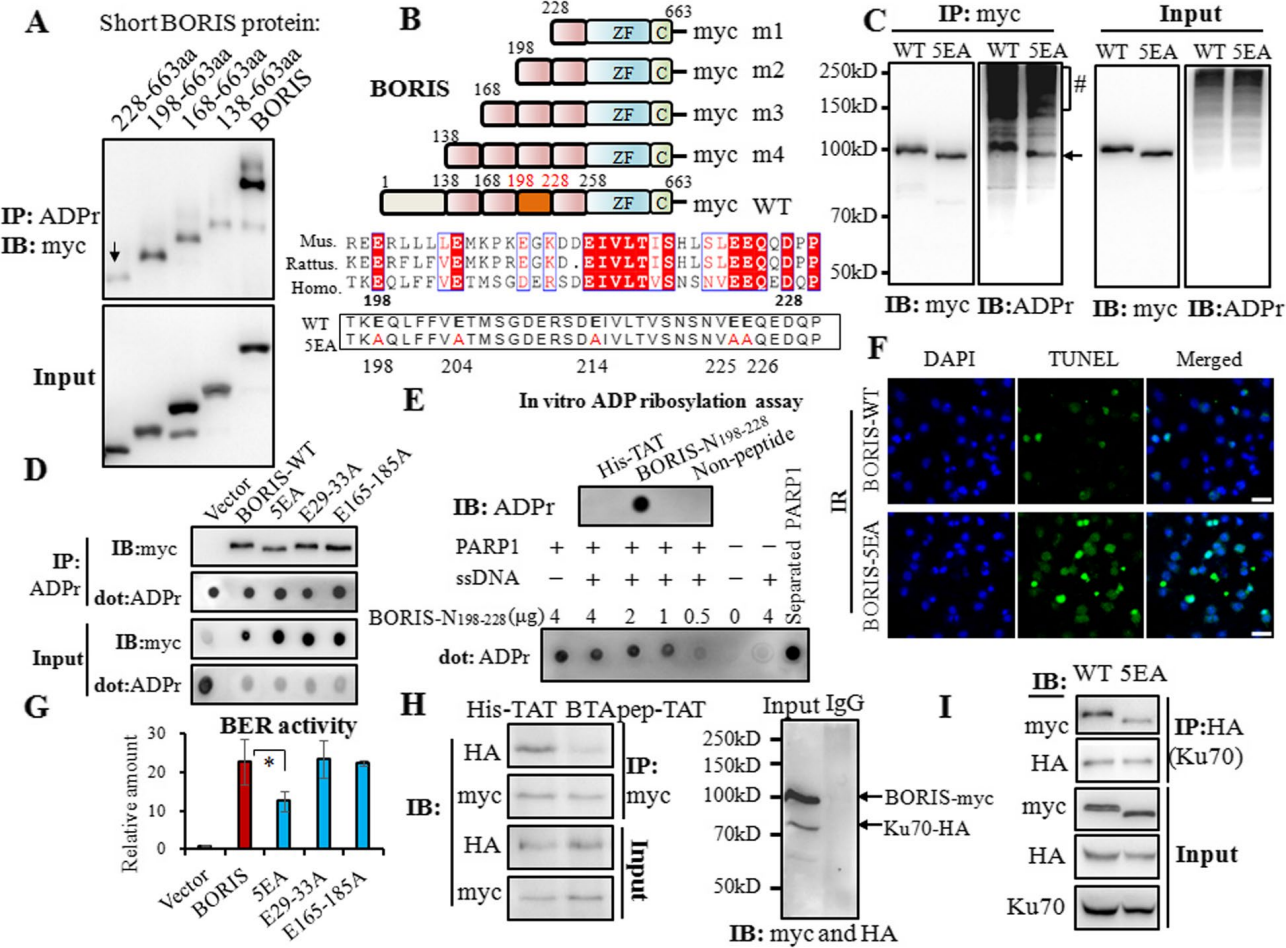
BTApep-TAT inhibited the ADP ribosylation at residues 198–228 of BORIS and inhibited the interaction of BORIS with Ku70. (**A**) Truncations of variable lengths of amino acids between AA 2 and 227 in the N-terminus of BORIS were constructed and transfected into H1299 cells. ADP ribosylation of the short BORIS protein, which contains residues 228–663 of BORIS, was reduced compared with that of BORIS 198–663. (**B**) Schematic representation of the truncated BORIS proteins and the details of the homology of BORIS in Mus musculus (Mus.), Rattus norvegicus (Rattus.) and Homo sapiens (Homo.). Conserved amino acids are shown in red boxes. Five conserved glutamic acid residues were mutated to alanine residues in the BORIS-5EA mutant. (**C**) The 5EA quintuple mutant of BORIS tagged with myc (5EA) or BORIS-myc (WT) was transfected into H1299 cells. The cell lysates were used to evaluate the interactions of ADP-ribosylated and myc-tagged proteins. (**D**) ADP-ribosylation of the 5EA quintuple mutant and other mutants (which contained substitutions of glutamic acid residues with alanine residues in the 29–33 or 165–185 regions) were compared by precipitation with an anti-ADP-ribose antibody and visualization by dot blot or Western blot assays with a myc antibody. (**E**) The BORIS-N_198-228_ peptide was subjected to PARP1-mediated PARylation in vitro, which was activated by recombinant PARP1 and ssDNA. After the reaction, the peptides and PARP1 were separated by centrifugal filtration and analyzed by the dot blot assay. (**F**) DNA damage induced by 30 Gy X-ray irradiation was examined in BORIS-WT- and BORIS-5EA-transfected H1299 cells by TUNEL assay. (**G**) The comparison of BER activity of the mutants is presented as a histogram. (**H**) BORIS-myc and Ku70-HA were co-transfected into H1299 cells, and the cells were incubated with BTApep-TA or His-TAT to investigate the interaction between BORIS and Ku70. (**I**) BORIS-myc/BORIS-5EA-myc and Ku70-HA were co-transfected into H1299 cells, and the interaction between BORIS and Ku70 was examined by immunoprecipitation

Since BTApep-TAT binds to BORIS-N_1-258_, interferes with ADP ribosylation of BORIS and suppresses BORIS-mediated DNA damage repair, we hypothesized that ADP ribosylation of BORIS-N_1-258_ was required for its function. To determine the sites of ADP-ribosylation in BORIS, we generated several truncation mutants. Deletion of AA 2–227 generated a truncated BORIS protein corresponding to AA 228–663, and ADP ribosylation was significantly reduced in this truncated product compared with that in AA 198–663 BORIS, which was generated by deleting AA 2–197. This observation suggested that the major site of modification is located between residues 198–228 (Fig. 5A and B). *ADPredict* (http://www.adpredict.net/) was used to predict the regions and sites in the N region of BORIS (Supplemental Fig. 4). The glutamate proximal sequence is known to be ADP-ribosylated [34]. According to the prediction by *ADPredict* [35], the glutamic acid residues 198, 204, 214, 225, and 226 were the putative ADP-ribosylation sites, that are evolutionally conserved among mouse, rat and human [36] (Fig. 5B). Therefore, we generated a quintuple mutant (5EA) of BORIS by replacing all five glutamic acid (E) residues within this region with alanine residues (A) (Fig. 5B). The 5EA mutation reduced ADP ribosylation of BORIS (Fig. 5C). Although the 5EA mutation did not completely abolish ADP ribosylation of BORIS, a significant decline was observed. Another two potential ADP-ribosylation regions (AA 29–33 and AA 165–185) within BORIS-N_1-258_ were also mutated by replacing the glutamic acid residues with alanine residues; however, these mutations did not attenuate ADP-ribosylation (Fig. 5D). As shown in Fig. 5C and D, apparent molecular weight was reduced with 5EA mutant but not with E29-33A or E165-185 mutants. Consistent with these observations, 5EA mutant was less active in DNA repair as demonstrated in Fig. 5F and I.

To further confirm ADP ribosylation at residues 198–228, the synthesized BORIS-N_198-228_ peptide was subjected to a PARP1-catalyzed in vitro PARylation assay. Single-strand DNA (ssDNA) was added to activate PARP1. The BORIS-N_198-228_ peptide was separated by centrifugal filtration, and its PARylation levels were examined by dot blotting using an anti-ADPr antibody. PARylation of the BORIS-N_198-228_ peptide accumulated in proportion to its abundance (Fig. 5E). To determine the modification between residues 198–228, the BORIS-N_198-228_ peptide was pre-incubated with BTApep-TAT or His-TAT before processing for PARP1-catelyzed in vitro PARylation. BTApep-TAT but not His-TAT inhibited PARylation (Supplemental Fig. 5A). This result indicated that BTApep-TAT inhibits PARylation at residues 198–228. BORIS-5EA was functionally inactive for DNA repair under irradiation in vivo (Fig. 5F) or in DNA ligation assays in vitro (Fig. 5G). When cells were incubated with BTApep-TAT, the interaction between BORIS and Ku70 was blocked (Fig. 5H). BORIS-5EA only weakly interacted with Ku70, suggesting that ADP ribosylation of BORIS was required for the binding between BORIS and Ku70 (Fig. 5I). Taken together, we provided evidence that ADP ribosylation of BORIS was responsible for the association with Ku70 and DNA repair. BTApep-TAT attenuated the function of BORIS in DNA damage repair by interfering with ADP ribosylation of BORIS and subsequent interaction with Ku70.

### BTApep-TAT inhibited the progression of subcutaneous tumors

**Fig. 6:**
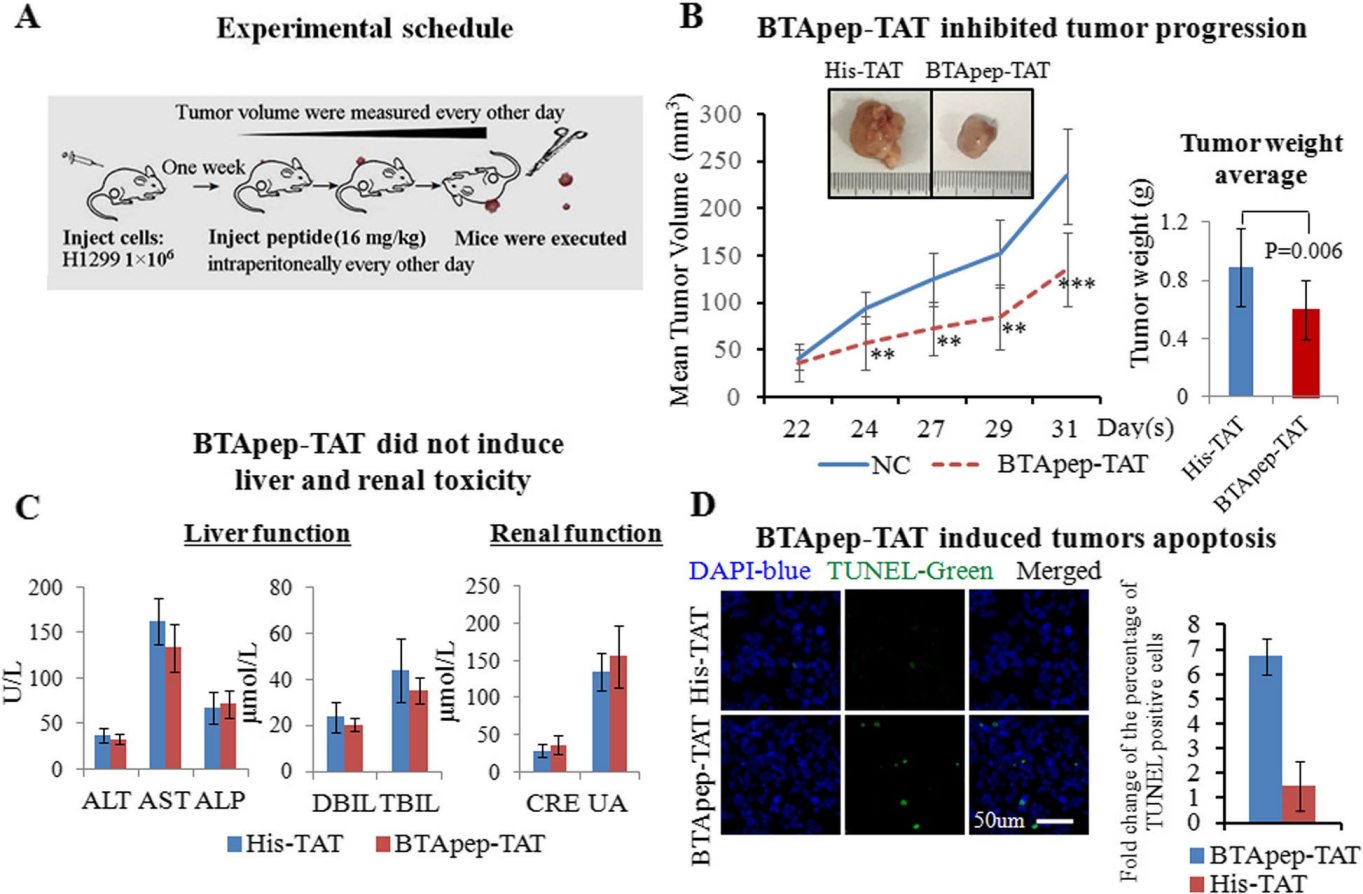
BTApep-TAT inhibited the progression of xenograft tumors. (**A**) The experimental schedule of the xenograft tumor and treatment experiments. (**B**) The growth of the NSCLC xenograft in animals treated with BTApep-TAT was compared with those treated with His-TAT. The left panel shows the changes in the tumor volume during the observation and treatment period. The right panel presents the differencee in the tumor weight between the two treatment groups at the end of the experiment. (**C**) Hepatic and renal functions after peptide treatments were examined by serum-based tests. (**D**) The extent of DNA damage was evaluated by TUNEL assays. The left panel shows representative images of the TUNEL assays captured by microscopy, and the right panel shows a statistical summary of the comparison of TUNEL-positive cells between the treatment groups

To test the function of BTApep-TAT in vivo, H1299 cells, that were sensitive to BTApep-TAT treatment in vitro, were used to generate a xenograft model in NOD/SCID/γc null (NSG) mice. H1299 cells (1 × 10^6^/injection) were injected subcutaneously under the forelimbs of 12 mice. After one week, mice were divided into 2 groups (6 mice per group) and intraperitoneally injected with 16 mg/kg BTApep-TAT or His-TAT every other day for 3 weeks. The tumors were evaluated every other day. The tumor weight was measured at the end of the experiment after euthanasia and tumor resection (Fig. 6A). Treatment with BTApep-TAT inhibited tumor growth compared with His-TAT treatment (Fig. 6B). Moreover, BTApep-TAT treatment did not induce liver or kidney toxicity (Fig. 6C). Treatment with BTApep-TAT also did not affect the body weight of the animals (Supplemental Fig. 5B). The tumors were sectioned and examined by a TUNEL assay. Three weeks of treatment with BTApep-TAT, but not with His-TAT, induced DNA damage in cells of the subcutaneous tumors (Fig. 6D).

## Discussion

BORIS was discovered 20 years ago and is well known to promote cancer progression [5]. However, compounds or inhibitors directly targeting BORIS have not been reported. In the present study, the inhibitory effects of BTApep-TAT were demonstrated by its interaction with BORIS, inhibition of the gene expression profile, and suppression of cancer cell proliferation and tumor growth. BTApep-TAT is a useful reagent to investigate BORIS function and could be efficacious in clinical application. BTApep-TAT will help to discover the interaction partners of BORIS, solve the structure of BORIS, and design small molecular weight compounds to inhibit BORIS. Proteolysis-targeting chimeras (PROTACs) are compounds used to degrade target proteins by using ubiquitin-protein ligase-conjugating compounds that recognize a target protein. Although the mechanism by which BTApep-TAT inhibited cancer progression was not determined clearly because the function of BORIS was not thoroughly investigated, the specificity of the BTApep-TAT interaction with BORIS may suggest that the formation of PROTACs induced BORIS degradation.

There were two studies that selected cytotoxic T lymphocytes targeting BORIS sf6 by using a specific amino acid sequence from the C-terminal region as an antigen. The strategy proved the feasibility of targeting BORIS sf6 for cancer therapy [2, 12]. BORIS sf6 shares the same N-terminus with the majority of other BORIS members but has a unique C-terminus and lacks zinc fingers 6–11 [13]. Antibodies used for BORIS detection in most studies were produced against the antigens located within the N-terminus amino acids 1–258. BORIS, but not BORIS sf6, has been reported to be frequently expressed in carcinoma. BORIS siRNA OCM-8054 [37], which targets the region between zinc finger 10 and 11 of BORIS, induces apoptosis of colorectal and breast cancer cells [17, 37,17, 24]. However, BORIS sf6 lacks the sequence targeted by OCM-8054. Treatment strategies designed to target BORIS sf6 showed only marginal effects on cancers. In addition, BORIS has not been reported to be expressed on the cell membrane. Additional studies are needed to determine whether intracellular BORIS can be efficiently recognized by the immune system. In our present study, we used BORIS AA 1–258 as an antigen for the selection of BORIS-targeting peptides because the N-terminal sequence AA 1–258 is present in the majority of the BORIS family members including sf6 [13]. The N-terminus of BORIS AA 1–258 will be more advantageous than the C-terminus of the BORIS sf6 form for designing therapeutic strategies based on BORIS.

BTApep-TAT considerably, but not very vigorously, suppressed NSCLC progression in a xenograft animal model in the present study. This could be due to the instability of the BTApep-TAT peptide. Treatment by the intraperitoneal injection in the present study may have contributed to BTApep-TAT degradation. We chose to administer BTApep-TAT by intraperitoneal injection because we considered that certain unpredictable problems, such as cytokine storm and toxicity, might be caused by subcutaneous injection of the peptide. Protective modifications of BTApep-TAT may increase peptide stability and persistence in the circulation. An understanding of the accurate structure of BORIS or the complex of BORIS and BTApep-TAT will help with the design of the modifications. Although the present study demonstrated the inhibitory effect of BTApep-TAT in an animal model, additional investigation is needed to evaluate whether this peptide can be used in clinics.

Double-strand DNA induced more ADP ribosylation of BORIS than ssDNA, while BORIS was apparently more efficient in SSBR than in NHEJ-directed DSB repair. Three reasons may explain these results. 1. The method used for NHEJ detection was based on gel electrophoresis, which cannot be quantified with sufficient precision. Quantitative real-time PCR may be more suitable for NHEJ evaluation. 2. The conditions, including temperature and reaction time, used for NHEJ ligation in vitro may have been suboptimal. 3. The precise mechanism of ADP ribosylation of BORIS is unknown. BORIS underwent both poly- and mono-ribosylation and this was mediated by PARP1, PARP4, PARP5a, PARP8, PARP13 and PARP16. PARP family members are responsible for the transfer of ADP-ribose from nicotinamide adenine dinucleotide (NAD +) to nuclear proteins and provide an ADP-ribose chain platform for mediating the rapid recruitment of DNA repair factors for SSB and DSB repair [38, 39] PARylation is involved in diverse biological processes, such as DNA replication, cell cycle regulation, and chromatin remodeling [40–43].Mono ADP ribosylation (MARylation) is involved in protein inactivation, viral immunity, and the cellular stress response [44–47]. In-depth understanding of the role of PARylation and MARylation of BORIS may inform the mechanisms of DNA repair mediated by BORIS.

As BORIS is ADP-ribosylated and is important for cancer cells, we considered that the PARylation inhibitor olaparib might inhibit BORIS. However, olaparib did not inhibit the ADP ribosylation of BORIS. Seventeen PAPRs have been known to belong to the PARP family [48–50], but olaparib inhibits only PARP1, PARP2 and, to a lesser extent, PARP3 [33, 49]. Based on our observation that BORIS was catalyzed by PARP4, PARP5a, PARP8, PARP13 and PARP16, it is expected that olaparib could not abolish ADP ribosylation of BORIS. Considering the importance of ADP ribosylation on the function of BORIS and the complex catalysis mechanism by multiple PARPs, the inhibition of BORIS by PARP inhibitors for cancer treatment will not be logical or efficient. Targeting the functional ADP-ribosylation sites of BORIS by specific BORIS inhibitors will be a rational and practical approach.

Timely recruitment of PARPs and Ku70/80 complex in responses to DNA damage is essential for the maintenance of genomic stability in normal cells [51, 52]. The recruitment and accumulation of Ku70 at the site of DNA double-strand breaks promotes the involvement of DNA repair factors for non-homologous end joining [51–57]. As Ku70 directly regulates DNA ligation without the need for a homologous template, the repair leaves mutations at the site and is inaccurate. BORIS is generally silenced in normal cells and only expressed in cancer cells, and the interaction of BORIS with Ku70 leads to unusual DNA repair in cancer cells. BORIS is the paralog of CTCF and binds to CTCF-like sites in the genome but is functionally different from CTCF [14]. CTCF organizes genomic DNA, colocalizes with the cohesin complex, regulates DNA replication and is essential for safeguarding the genome [58–60]. The abnormal DNA repair regulated by BORIS in cancer cells might disrupt the genome stability established by CTCF. Genome instability is a hallmark of cancer. The inappropriate responses to DNA damage or DNA repair disorders are linked to rearrangement of the genome, microsatellite instability, chromosome instability, and tumorigenesis [61, 62]. We found that BORIS was ADP-ribosylated within 30 min after stimulation with single- and double-strand DNA, and BORIS facilitated the recruitment of Ku70. These findings suggest that direct participation of BORIS in the DNA repair complex may disrupt normal DNA repair process in cancer cells. The BORIS inhibitor BTApep-TAT may revolutionize our ability to regulate the functions of BORIS, including posttranslational modifications and interaction with its binding partners. The competition between PARP1 and the Ku complex at DSBs may play an important role in the choice of the DSB repair pathway [26, 39]. Further research is required to understand the composition of the complex recruited by ADP-ribosylated BORIS [26, 51, 52].

## Conclusion

The present study demonstrated that BTApep-TAT peptide could target BORIS. BTApep-TAT interacted with BORIS and suppressed BORIS-associated transcriptomes, mimicking the effects of *BORIS* knockdown. BTApep-TAT suppressed cancer cell proliferation, induced cancer cell apoptosis, inhibited NSCLC progression in a xenograft model, and suppressed ADP-ribosylation of BORIS. ADP-ribosylation of BORIS at residues 198–228 contributed to the interaction with Ku70 and was responsible for DNA repair. Our results suggest the feasibility of using BTApep-TAT peptide for cancer therapy and provide a basis for future study to dissect the mechanism of the regulation of BORIS and to design optimized inhibitors of BORIS.

## Supplementary Information

**Additional file 1.** Supplementary table 1-5 and Supplementary figures 1-6

**Additional file 2.** Supplementary table 6

## Data Availability

RNA-sequencing data are available. The BioProject accession number is PRJNA832514.

## Abbreviations

BORIS: Brother of regulator of imprinted sites
NSCLC: Non-small-cell lung cancer
PTM: Post translational modification
TCGA: The Cancer Genome Atlas
CTL: Cytotoxic T cell
CTCF: CCCTC binding factor
UTRs: Untranslated regions
BTApep: BORIS-targeted peptide
BORIS-N_1-258_: BORIS N-terminal region 1-258AA
BORIS-del N_1-258_: BORIS N-terminal deletion
BLI: Biolayer interferometry
Kd: Binding affinity, dissociation constant
ADPr: ADP ribosylation
IR: Irradiation
ROS: Reactive oxygen species
SSBs: DNA single-strand breaks
DSBs: Double-strand breaks
SSBR: Single-strand break repair
BER: Base excision repair
NHEJ: Nonhomologous end joining
HDR: Homology directed repair
dsDNA: Double-strand DNA
ssDNA: Single-strand DNA
NSG: NOD/SCID/γc null mice
PROTACs: Proteolysis-targeting chimeras
PDX: Patient-derived xenograft
NAD +: Nicotinamide adenine dinucleotide
MARylation: Mono ADP ribosylation
PARylation: Poly ADP ribosylation
