# Supplementary material for: BTApep-TAT peptide inhibits ADP-ribosylation of BORIS to induce DNA damage in cancer
Source: Mol Cancer. 2022 Aug 2;21:158. doi: 10.1186/s12943-022-01621-w (PMC9344678; doi:10.1186/s12943-022-01621-w)
Supplement: Supplementary file 1 — Additional file 1. Supplementary table 1-5 and Supplementary figures 1-6 [file 12943_2022_1621_MOESM1_ESM.docx]

*Supplementary data*

**BTApep-TAT peptide inhibits ADP-ribosylation of BORIS to induce DNA damage in cancer**

The supplementary file includes 6 Figures and 6 Tables.

**Supplemental Table Ⅰ. Source of the plasmids used in this study**

| Plasmids | SOURCE |
| --- | --- |
| BORIS-myc PCMV6 plasmid | Origene |
| PCMV6 Vector | This paper |
| pFN6K | Promega |
| BORIS –N _1-258_-PCMV6 | This paper |
| BORIS -N _1-258_-pFN6K | This paper |
| BORIS -del N-PCMV6 | This paper |
| BORIS -del N-pFN6K | This paper |
| BORIS-RFP | This paper |
| BORIS- N _1-258_-his PCMV6 | This paper |
| BORIS 228-663aa | This paper |
| BORIS 198-663aa | This paper |
| BORIS 168-663aa | This paper |
| BORIS 138-663aa | This paper |
| HA-Ku70 Donated by Jing Jia of Hangzhou medical college | |

**Supplemental Table Ⅱ. The sequence of siRNAs**

| siRNA/Oligos | Sequences (5’to3’) |
| --- | --- |
| siBORIS-1 | GGAAAUACCACGAUGCAAA |
| siBORIS-2 | GGAUCAACCUACAGCUGGU |
| siPARP1 | GCCUCCGCUCCUGAACAAU |
| siPARP2 | AAUCAGUGUAAUGAACUACUA |
| siPARP3 | GGACCCAGGUGUAUGAGGACUACAA |
| siPARP4 | AAACAAGGAUUUCUACUAAGA |
| siPARP5a | CAGUAACAAUUCACCGUCGUCCUCU |
| siPARP5b | GCUUCAGAAUGGUGCAAAU |
| siPARP7 | GUGAUAAGCUGAGUACUGATT |
| siPARP8 | GGAAGAUUCUGAAGGUGACAAUGAU |
| siPARP10 | GCCUGGUGGAGAUGGUGCUAUUGAU |
| siPARP13 | GCUCACGGAACUAUGAGCUGAGUUU |
| si-( Negative siRNA) | UUCUCCGAACGUGUCACGUdTdT |

**Supplemental Table Ⅲ. The sequence of primers**

| Primers | Sequence (5’to3’) |
| --- | --- |
| BORIS -N terminal-F | GGAACCTTCCACCCGCTCGAGCAGAAACTC |
| BORIS -N terminal-R | TCGAGCGGGTGGAAGGTTCCTTTTGCTCCC |
| BORIS-del N-F | GCGATCGCCATGTTCCACTGTGATGTCTGCATGTTC |
| BORIS-del N-R | ATCACAGTGGAACATGGCGATCGCGGCGGCAGATCT |
| BORIS- N _1-258_-his-F | GAAAACCTCTACTTCCAGGGACATCATCACCATCACCATTAAACGGCCGGCCGCGGTCA |
| BORIS- N _1-258_-his-R | ATGGTGATGGTGATGATGTCCCTGGAAGTAGAGGTTTTCGGTTCCTTTTGCTCCCTTTG |
| BORIS 228-663aa-F | GCCGCGATCGCCATGGATCAACCTACAGCTGGTCAAGCAG |
| BORIS 228-663aa-R | CATGGCGATCGCGGCGGCAGATCTCCTCGG |
| BORIS 198-663aa-F | GCCGCGATCGCCATGCAGCTCTTTTTTGTGGAAACAATGTCAGG |
| BORIS 198-663aa-R | CATGGCGATCGCGGCGGCAGATCTCCTCGG |
| BORIS 168-663aa-F | GCCGCGATCGCCATGTTAGCGGTGAGCCTGGCTGAAACTG |
| BORIS 168-663aa-R | CATGGCGATCGCGGCGGCAGATCTCCTCGG |
| SSBR Primer 1 | ATGGCGGGGCTCTCCAGAACATCATCAATTCCCGGACGTCTAAACCAAACCACTTTCAC |
| SSBR Primer 2 | P-AGGTCCAGGTCTGGAAGGCTGTGGGCAAGGTCATAT |
| SSBR Primer 3 | ATGGCGGGGCTCTCCAGAACATCATCAATTCCCGGACGTCTAAACCAAACCACTTTCA |
| SSBR Primer 5 | CCAGACCTGGACCTGTGAAAGTGGTTTGGTTTAGACGTC (ddC) |
| SSBR Primer 6 | ATGGCGGGGCTCTCCAGAACATCGTCCGGGACCTGACTGACTACCTCATGAACAGGTCCAGGTCTGGAAGGCTGTGGGCAAGGTCATAT |
| SSBR Primer 7 | CCAGACCTGGACCTGTTCATGAGGTAGTCAGTCAGGTCCCGGAC(ddC) |
| SSBR Forward-S | CGGGGCTCTCCAGAACATC |
| SSBR Reverse-AS | ATGACCTTGCCCACAGCCT |
| SSBR Probe 1 | FAM-CAATTCCCGGACGTCTAAACCAAACCACTTTC-TAMRA |
| SSBR Probe 2 | HEX-CCGGGACCTGACTGACTACCTCATGA-FAMRA |

**Supplemental Table Ⅳ. Antibodies and materials that applied in immunotechnique**

| Antibodies | SOURCE | IDENTIFIER |
| --- | --- | --- |
| Rabbit monoclonal Poly/Mono-ADPr | Cell Signaling Technology | Cat#83732 |
| Mouse monoclonal anti-Myc-Tag | Cell Signaling Technology | Cat#2276 |
| Rabbit monoclonal anti-His-Tag | Cell Signaling Technology | Cat#12698 |
| Streptavidin magnetic bead conjugate | Cell Signaling Technology | Cat#5947 |
| Poly-ADPr | Trevigen | Cat#4335-MC-100 |
| Mono-ADPr | Sigma-Aldrich | Cat#MABE1076 |
| Rabbit polyclonal anti-GAPDH | Sigma-Aldrich | Cat#G9545 |
| Mouse IgG | Sigma-Aldrich | Cat#I5381 |
| Rabbit IgG | Sigma-Aldrich | Cat#I5006 |
| Mouse monoclonal anti-BORIS | Santa Cruz Biotechnology | Cat#sc-377085 |
| Protein G PLUS-Agarose | Santa Cruz Biotechnology | Cat#sc-2002 |
| Protein A Agarose | Santa Cruz Biotechnology | Cat#sc-2001 |
| HRP conjugated anti-M13 antibody | GE healthcare | Cat#27-9421-01 |
| Tubulin | HuaBio | Cat#ET1602-4 |
| DAPI | Beyotime | Cat#C1002 |

**Supplemental Table Ⅴ. Chemicals and reagents in this study**

| Chemicals, Assays and Reagents | SOURCE | IDENTIFIER |
| --- | --- | --- |
| Phage display peptide library Kit | New England Biolabs | Cat#E8111 |
| Biotin | Thermo Fisher | Cat#20217 |
| Lipofectamine 2000 | Thermo Fisher | Cat#11668019 |
| TRIzol reagent | Thermo Fisher | Cat#15596026 |
| opti-MEM | Thermo Fisher | Cat#31985070 |
| RNAiMAX | Thermo Fisher | Cat#13778075 |
| SYBR Gold I | Thermo Fisher | Cat#S11494 |
| Fast Mutagenesis System | Transgen | Cat#FM111-01 |
| BCA protein assay kit | Biosharp | Cat# BL521A |
| SDS-PAGE loading buffer | Biosharp | Cat# BL502B |
| DMSO | Sigma-Aldrich | Cat#D2650 |
| TMB | Sigma-Aldrich | Cat#T8665 |
| BSA | Sigma-Aldrich | Cat#V900933 |
| Cocktail | Roche | Cat#04693132001 |
| Biotin-PAR polymer | Trevigen | Cat#4336-100-02 |
| PARP Enzyme | R&D Systems | Cat#4668-100-01 |
| WB Sirius ECL | Advansta | Cat#k-12043-D10 |

**Supplemental Table VI is shown in a separate Excel file.**


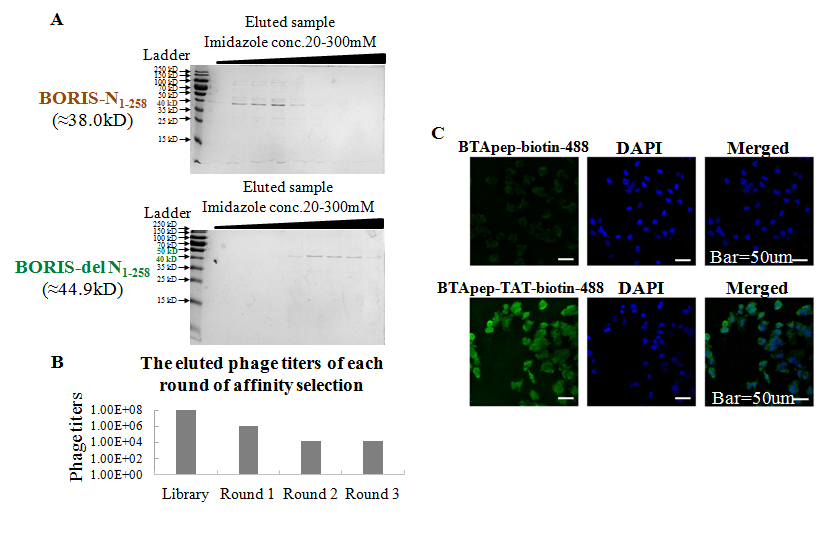


**Supplemental Figure 1. Selection and characterization of BORIS-binding peptides.** (A) The BORIS-N_1-258_ and BORIS-del N_1-258_ proteins were purified by Ni-NTA column chromatography and detected by anti-His antibody by western blotting. (B) The enriched phage clones that contained individual peptides were examined using BORIS-N_1-258-_coated plates by ELISA. Enrichment of eluted phages during each round was examined. (C) BTApep-TAT-biotin was compared with BTApep-biotin to test the cell membrane permeability. Alexa Fluor® 488-labeled streptavidin was used to detect the biotin-conjugated peptides.


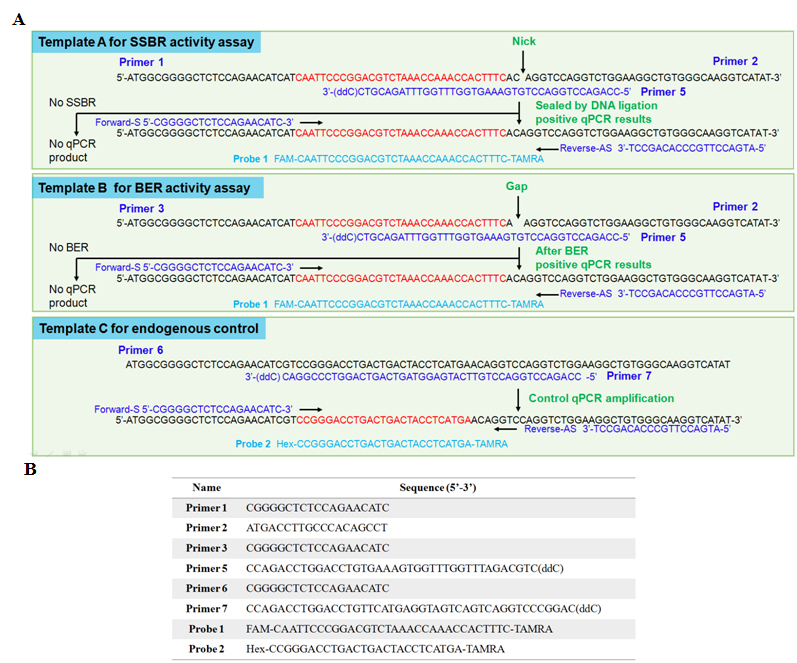


**Supplemental Figure 2. SSBR and BER assays.** (A) The target templates of DNA break fragments, nick sites and PCR primers and probe used for the detection of DNA ligation are listed on top. The target templates of DNA break fragments with single nucleotide deletion and PCR primers and probe used for the detection of DNA ligation are listed in the middle. The intact control template without breaks is listed at the bottom. (B) The primers and probes used to detect the ligation efficiency of the target templates are listed in the table.


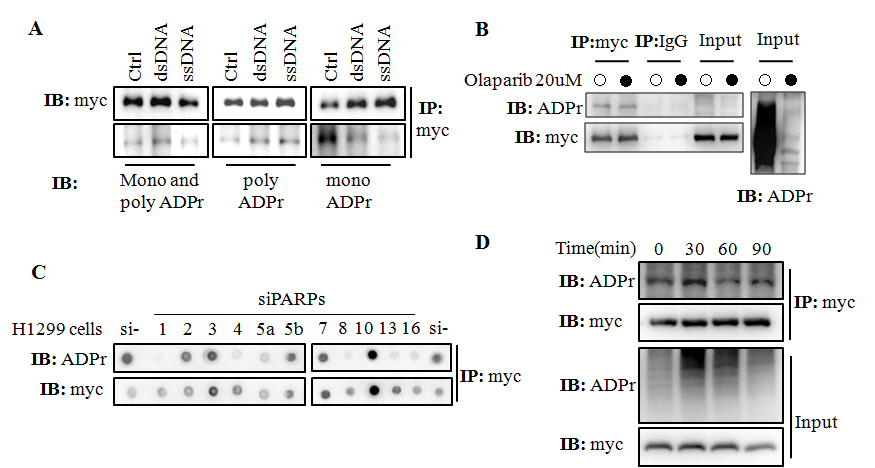


**Supplemental Figure 3. Detection of ADP modification of BORIS and the PARPs that catalyze the ADP modification of BORIS.** (A) Poly-ADPr and mono-ADP-ribosylation were distinguished by using antibodies specific for either poly-ADPr or mono-ADP-ribose. DNA damage was presented as artificial dsDNA or ssDNA. (B) Olaparib strongly inhibited ADP ribosylation on PARP but did not inhibit ADP ribosylation of BORIS. (C) PARP knockdown was performed by siRNAs individually. Knockdown of *PARP1*, *PARP4*, *PARP5a*, *PARP8*, *PARP13* and *PARP16* inhibited ADP ribosylation of BORIS. Among those PARPs, PARP1 and PARP5a generate poly-(ADP-ribose); however, PARP4, PARP8, PARP13 and PARP16 generate mono-(ADP-ribose). (D) A mixture of single- and double-stranded DNA was used to stimulate the ADP ribosylation of BORIS for 30, 60, and 90 minutes. ADP-ribosylation peaked at 30 minutes and declined after 60 minutes.


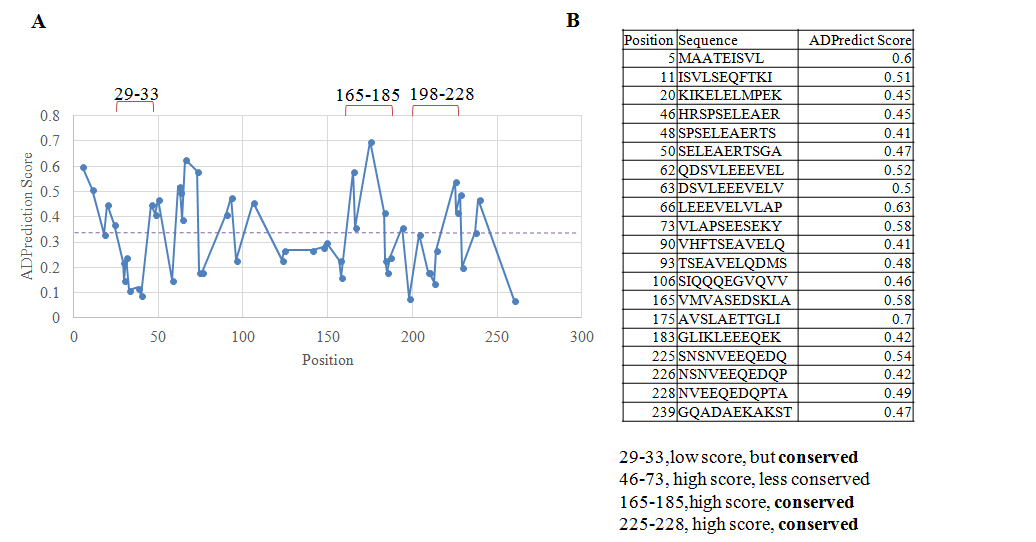


**Supplemental Figure 4**. **The prediction of the N section of BORIS by *ADPredict* (http://www.adpredict.net/).** Four core ADP-ribosylation regions are presented by diagram and peptide sequences. The three conserved regions are AA 29-33, AA 165-185, and AA 225-228.


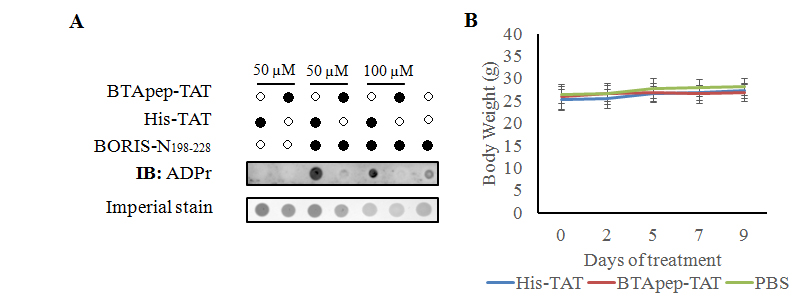


**Supplemental Figure 5**. **The effect of BTApep-TAT on the PARylation of the BORIS-N_198-228_ peptide in vitro and the animal body weights under BTApep-TAT treatment.** (A) The BORIS-N_198-228_ peptide was pre-mixed with BTApep-TAT or His-TAT before being subjected to PARP1-mediated in vitro PARylation. BTApep-TAT but not His-TAT inhibits PARylation. Our data demonstrate that BTApep-TAT inhibits PARylation of the BORIS-N_198-228_ peptide in vitro. (B) Treatment with BTApep-TAT did not affect the body weight of the animals.


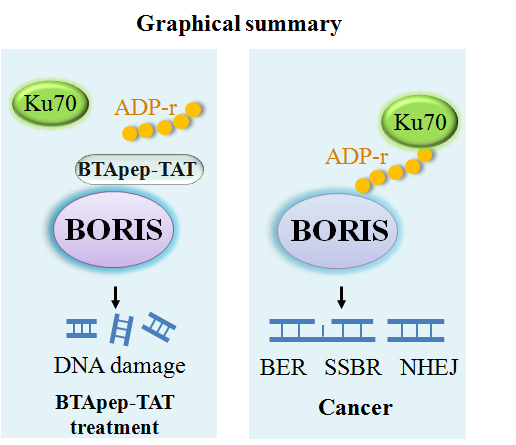


**Supplemental Figure 6.** Graphical summary for this work.
